# 

**Published:** 1811-06

**Authors:** 


### MONTHLY CATALOGUE OF MEDICAL BOOKS.

Cursory Remarks on Contagious Diseases and on Baths. By M. L. Este, Esq. late Lecturer on Animated Nature, and the Philosophy of the Animal Economy, at the Royal Institution of Great Britain, &c. &c. 2 parts, 8vo. Callow.

A Letter concerning the Royal and other scientific Institutions; respectfully addressed to their managers, proprietors, and subscribers. By M. L. Este, Esq. 8vo. Callow.

Communications relative to the Datura Stramonium, or Thorn-apple, as a cure or relief of atshma; addressed to the Editor of the Monthly Magazine. Several of them never before published. With a coloured plate, 8vo. Phillips.

Surgical Observations on Tumours and on Lumbar Abscesses. By John Abernethy, F.R.S. 8vo. 4 Vol. Longman and Co.

Disquisitions in the History of Medicine; part first, exhibiting a view of Physic, as observed to flourish, during remote periods, in Europe, and the East. By Richard Millar, M. D. Lecturer on Materia Medica in the University of Glasgow, &c. 8vo. Murray.

### NOTICES TO CORRESPONDENTS.

Communications from H. E. Clough, M. D. Messrs. Fordham, M'Culloch, Harrold, Philanthropos, Obstetricus, &c. &c. &c. have been received, which will appear in due course.

The conclusion of the paper on Stramonium will appear in our next: besides other matter, it will contain some interesting practical information, with cases illustrating the effects of smoking that plant in asthma, by Dr. Bree, Dr. Gooch, &c.

The Title page and Index to this Volume will be given in the next Number.

### INDEX.

ABSORPTION, on the organs of 44
Abstinence, cases of 85
Acid, agency of acetous 333
—— on the muriatic 446
Adams's, Dr. edition of Hunter on the venereal disease 80
Æther, its use in strangulated hernia 28
Agaricus, on different species of 123
Agricultural prize questions 455
Air, its effects on meat 252
Albicore, poisonous qualities of the 398
Alconorque, observations on 546
Aleppo, on the scammony of 157
Allanite, a new metal, described 181
Amaurosis, case of 510
Andrews, Dr. on an enlarged lower extremity 400
Aneurism, cases of 356
Aneurismal needle, on the invention of the 32, 238
Animals, on luminous 409, 512
Anus, case of preternatural 449
Apoplexy, on the treatment of 229
Ardusset, Dr. on hernia 28
Arnold, Dr. on diseases of the troops 13
Arran, on the rocks of the island of 453
Arsenical solution in hooping cough 115
Artery, case of obliterated iliac 35
Ascites, case resembling 164
—— use of tobacco in 233
Asthma, on stramonium in 66, 377
—— on tobacco in 231
Atmosphere, variations of the 53
—— influence of the moon on the 85
—— on the changes of the 144
Bailey, Mr. on the eau medicinale 114
Baird's, Dr. case of carditis 396
Barlow's, Mr. case of small pox 390
Barometer, on the 52, 149
Barytes, a test for adulterated vinegar 85
Bateman, Dr. on the larvæ of insects 446
Bath, on the vapour 317
Batham's, Dr. case of repletion 475
Baxter's, Mr. case of excision of the uterus 210
Beddoes, Dr. memoirs of 91, 172, 259, 342
Beet-root, sugar extracted from 363
Berne, account of hospitals at 407
Beroe fulgens described 414
Bernoully, M. on potass 518
Berthollet, M. on the effects of the air 252
Bladder, rupture of the gall 185
Blair, Mr. on syphilis 364
Blear-eye, treatment of the 85
Bleeding, case of copious 31
Blood, cause of the red colour in the 281
—— on white serum of the 152, 300
—— root, description of 166
Bones, an article of commerce 98
BOOKS, CRITICAL ANALYSIS OF MEDICAL.——Transactions of the Medical Society of London, 56, 161. Dr. Domeir on the Climate, Manners, &c. of Malta, 64. Fisher's Treatise on the Asthma, 66. Stevenson on the morbid sensibility of the Eye, 67. Hunter's Treatise on the Venereal Disease, by Dr. Adams, 80. Freake on the Humulus, 169. Dr. Stock's Memoirs of Dr. Beddoes, 172, 259, 342. Ramsden's Observations on the Sclerocele, &c. 175, 265, 350 Faulkner on the establishment of foreign Hospitals, 348. The Edinburgh Medical and Surgical Journal, 357, 441. Dr. Gourlay's Observations on the Natural History, &c. of Madeira, 425. Dr. Bostock's Remarks on the Nomenclature of the New London Pharmacopœia, 431. Cursory Remarks on Corpulence, 494. Dr. Farrell on Ophthalmia, 522. Mr. Gibson on the artificial Pupil, 535.
Bostock, Dr. on the new Pharmacopœia 431
Botanical reports 92, 187, 370, 549
Bowels, case of affection of the 61
Braconnet, Mr. on resinous substances 320
Bradley, Dr. on puerperal fever 193
Bree, Dr. on asthma 231
Brodie, Mr. on muscular motion 180
—— on vegetable poisons 361
Brookes, Mr. on fossil remains 97

Brookes, Mr. on the white serum 130
Budding, new method of 253
Burns, Mr. on suppuration of the liver 164
Calculus, new species of 254
Calmucks, on the Koumiss of the 251
Calomel in dysentery, on 21
———— in obstinate vomiting, efficacy of 308
Caloric, agency of 328
Cancer, corrections in a case of 32
———— queries on 117
————fulgens described 411
Carditis, case of 396
Cartmell in Lancashire, medicinal spring at 84
Cases—Of fever, 2; dysentery, 7; tape-worm, 23; change of colour, 24; vicarious menstruation, *ibid.* strangulated hernia, 28; of twins, 29; of eating cherries, 31; copious bleeding, *ibid.* obliterated iliac artery, 35; fatal effects of white lead, 60; affection of the bowels, 61; bite of a cat, 61; elephantiasis, 84; scarlatina anginosa, 102; premature puberty, 117; white serum of the blood, 152, 300; suppuration of the liver, 164; tumours in the head, 166; tape worm, 167; gout, 170; rupture of the gall bladder, 185; epidemic puerperal fever, 193; fractured vertebræ, 201; hæmorrhage, 225; morbid enlargement of the clitoris, 236; child born without eyes, 277; derangement in a fœtus, 279; of sarcocele, 294; fungus hæmatodes, 303; obstinate vomiting, 308; singular case of twins, 311; use of the vapour bath, 319; of aneurism, 356; of embryulcia, 387; small pox, 390; of carditis; 396; of enlarged lower extremity, 400; malformation of the genital organs, 441; of gonorrhæa, 445; of fungus hæmatodes, 448; of præternatural anus, 449; of repletion, 475; of periodical spasm, 477; of amaurosis, 510.
Castor oil, its use in dysentery 22
Cat, case of the bite of a 61
Catarrh, prevalence of 82, 284
Ceylon, vaccination in 460
Charcoal, experiments with 457
Cherries, fatal effects of eating 31
Child, one born without eyes 277
Child-bed, proportion of deaths in 213
Chorea, on the treatment of 208
Christie, Mr. on vaccination 460
Clarke's, Dr. account of the Calmucks 251
Clitoris, morbid enlargement of the 236
Clough's, Dr. case of twins 29
———— of embryulcia 387
Colman, Mr. on glanders and farcy 281
Colour, extraordinary instance of change of 24
Conquest's, Mr. case of malformation of the genital organs 441
Cookson, Dr. on premature puberty 117
Cophosis, case of 510
Copland, Mr. on the muriatic acid 446
Corpulence, remarks on 449
Correspondents, notices to 283
Crocodile, a remarkable fossil 97
Cullen, Mr. on white serum 300
Cystic oxyde, on 254
Davies, Mr. on obstinate vomiting 308
Davy, Mr. on the chemical relations, &c. 83
——— his Bakerian lecture 180
——— on meteoric stones 545
Deering, Mr. on white lead 60
Devonshire, account of exotics in 243
Diseases of troops, on the 13
———— in London, reports of 82, 178, 283, 369, 548
———— of Mexico, on the 239
Division of medical practice 391
Dixon, Dr. on the bite of a cat 61
Dogs, on the sense of 137
——— account of the dissection of two 449
Domeier's, Dr. account of Malta 64
Dropsy, use of tobacco in 232
———— remarkable case of 234
Dublin, state of the college of surgeons at 89
Dysentery, cases of 3
———— in a long voyage, account of 37
Eagland's, Mr. truss for exomphalos 310
Eclipse of the sun, on an 453
Earle, Mr. on the aneurismal needle 32
———— his explanation 141
Eau medicinale, efficacy of 114
———— inquiries concerning the 183
———— observations on 184
———— fatal effects of 207
Edinburgh Journal, account of 357, 441
———— report of vaccine institution at 460
———— meetings of the Royal Society of 181, 362
Edmonstone, Dr. on hooping cough 359
Eggs, experiments on 278
Egypt, on the sarcocele of 289
Electricity, account of lectures on 280

Elephantiasis, case of 84
—— description of the 427
Embryulcia, case of 387
Epilepsy, remedy for 167, 456
Essex practitioner, on twin cases 485
Euphorbium, analysis of 324
Exomphalos, description of a truss for 310
Exotics in Devonshire, on 248
EXPERIMENTS—On the organs of absorption, 45; on the chemical relations, 83; on scammony, 156; on reddening litmus, 160; on metallic oxydes, 180, on meat, 252; on the blood of diabetic patients, 278; on vital heat, *ibid.*; on electrical, 280; on the red colour of the blood, 281; on James's powder, 316, on gamboge, &c. 320; on pus, 326; on the thoracic duct, 361; on zeolite, *ibid.*; on vegetable poisons, *ibid.*; on zuthine gas, 362; galvanic, *ibid.*; on muriatic acid, 446; on plants, 452; on potass, 456; on charcoal, 457.
Extremity, enlarged lower 400
Eye, on the morbid sensibility of the 67
—— on the treatment of blear 85
—— diseases of the 161
Eyes, case of a child born without 277
Faber's strychnomania, account of 385
Falconer, Dr. on mercurial preparations 61
Farrell, Dr. on ophthalmia 522
Farcy, observation on the 281
Faulkner, Dr. on foreign hospitals 348
Ferriar, Dr. on hooping cough 115
Fever, report on the Walcheren 1
—— on hectic 78
—— on an epidemic puerperal 193
Finsbury Dispensary, address to the 459
Fishes, on the poison of 398
—— on the luminous properties of 409
Fisher, Mr. on stramonium 66
Fœtus, singular malformation of a 279
Fogo, Mr. on twin cases 480
Foote, Mr. on imperfect perforation of the glans penis 41
—— reply to 118
Fossil remains, observations on 97
Fothergill's, Dr. A. case of tic douloureux 167
Fraxinella, on perspiration of 530
France, state of vaccination in 181
—— improvement of medical science in 454
Freake, Mr. on humulus 169
Fungus hæmatodes, on 303
—— case of 448
Gall bladder, rupture of the 185
Galvanic piles, on the powers of different 362
Gambogc, analysis of 320
Genital organs, malformation of the 441
Glanders, on inoculating 281
Glans penis, on too small a perforation of the 42
Glass, method of giving a red colour to 363
Glasgow, state of vaccination at 458
Golding's, Mr. case of parturition 205
Gonorrhœa, cases of 445
Good, Mr. on medical technology 56
Goodwin, Mr. on tape worm 23
Gourlay, Dr. on the climate of Madeira 425
Gout, on the French medicine for the 114, 183, 184, 207
—— efficacy of the humulus in 169
—— on savine in 544
Grass lands, new mode of improving 54
Greenland, description of new minerals from 181
Grimstone, Mr. on tetanus 359
Gums, analysis of resinous 156, 320
Hamilton, Dr. on the Walcheren fever 1
—— Mr. on scarlatina 101, 406
Hare lip, on the 165
Harrison, Dr. on eating cherries 31
—— on the proposed plan of 109, 216, 402, 492
Harrold's, Mr. case of fractured vertebræ 201
Hawkins, Mr. on exotics 248
Heat, on the nature of vital 278
Hectic fever, on 78
Hepatitis, ill effects of calomel in 62
Hernia, use of ether in strangulated 28
Hewson, William, memoirs of 59
Hill's, Mr. surgical remarks 487
Home, Mr. on the thoracic duct 361
Hooping cough, on arsenical solution in 115
—— plan for diminishing the 359
Horse-flesh eaten in Norway 283
Horses, treatment of pulmonary complaints in 281
Hospitals, on the establishment of 348
—— at Berne described 407
Humboldt, on the diseases of Mexico 239
Hufeland Mr. on savine 544
Humphries, Mr. on a disease of the eye 77
Humulus in gout, efficacy of 169
Hunter, John, on the venereal disease 78

Hunter, Dr. W. on the practice of 387
Hydrocele, on the acute 351
——— on the spurious 353
Hydrophobia, case resembling 61
——— observations on 235
Hydrophthalmia, observations on 163
Hydropic complaints, treatment of 6
Iceland, account of the hot springs of 181, 362
——— mineralogy of 362
Iliac artery, case of obliterated 35
India, fall of a meteoric stone in 457
Indians, on the American 243
——— remarkable superstition of 125
Infection from inoculated small pox 490
Inflammatory diseases, on 546
Insects, on the larvæ of 447
Intermittents, on the treatment of 4
Ipecacuanha in dysentery, use of 22
Ireland, Dr. on fungus hæmatodes 303
Jalap in dysentery, use of 22
James's powder, analysis of 315
James I. his antipathy to tobacco 121
Jenkins, Jeremiah, remarks on a pamphlet by 392
Jones's, Mr. case of twins 311
——— remarks on 386
Kentish, Dr. on the vapour bath 317
Kenworthy's, Mr. case of vicarious menstruation 25
Knight's, Mr. method of budding 253
——— remarks on the radicles of plants 452
Koumiss of the Calmucks, on the 251
Lafource, M. on amaurosis 510
Lancashire, medicinal spring in 84
Lands, new mode of improving grass 54
Langstaff, Mr. on luminous animals 416
——— on gonorrhæa 445
Larrey, M. on the sarcocele of Egypt 289
Larvæ of insects, on the 447
Leadbeater's, Mr. case of spasm 477
Lectures announced.—Mr. Shute on Anatomy, &c. 90. Dr. Buxton on the Practice of Medicine, *ibid.* Mr. Taunton on Anatomy, &c. *ibid.* Mr. Brookes on Anatomy, &c. *ibid.* Dr. Adams on the Institutes and Practice of Medicine, 91. Mr. Stevenson on the eye and ear, 96. At St. Thomas's and Guy's hospitals, 186. Dr. Watt on the Theory and Practice of Medicine, 186. Drs. Hooper and Agar on Physic, Chemistry, and the Materia Medica, *ibid.* Mr. Singer on Electricity, 280. Dr. Clough on Midwifery, 368. Dr. Reid on the Theory and Practice of Medicine, *ibid.* Mr. Taunton on Anatomy and Surgery, 461. At Guy's hospital, 462. M. Stevenson on the Eye and Ear, *ibid.* Dr. Adams on Medicine, 547. Mr. Thompson on Botany, *ibid.* Mr. Brande on Chemistry, *ibid.* Dr. Tuthill on Physic, *ibid.* Dr. Merriman on Midwifery, *ibid.* Drs. Hooper and Agar on Physic, *ibid.* Dr. Haighton on Midwifery, *ibid.* Mr. Brookes on Anatomy, *ibid.*
Leipsic fair, number of books at 283
Lepra Arabum, case of 84
Lewis's, Mr. case of morbid enlargement of the clitoris 236
Liver, case of suppuration of the 164
Liverpool, progress of vaccination at 304
London, transactions of the Medical Society of 56, 161
——— reports of diseases in 82, 178, 283, 369, 548
Luminous animals, observations on 409
Lyall, Mr. on staphyloma pellucidum conicum 359
——— on fraxinella 530
Lyons, prize question of the society of 454
Macartney, Mr on vital heat 278
——— on luminous animals 409, 434
Mackenzie, Sir George, his account of Iceland 181, 362
Madeira, description of the island of 425
Magnenus on tobacco 224
Magnesia, its use in dysentery 22
Majendie, Dr. on the organs of absorption 44
Malta, on the climate of 64
Mammoth, account of the 100
Mastiç, description of 160
Meat, alteration produced by air and water on 252
Medical Society of London, transactions of the 56, 161
——— proceedings of 364
——— and philosophical intelligence 88, 180, 277, 361, 452, 543
——— publications, catalogue of 91, 187, 369
——— technology, observations on 56
——— practice, on the division of 391
Medusa pellucens, description of the 411
Menstruation, case of vicarious 24
Mercury, process for making the muriate of 363

Mercury, on the fulminating powder of 457
Mercurial preparations, on the indiscriminate use of 61
Merriman, Dr. on an excision of the uterus 210
Meteorological tables and reports 95, 142, 191, 287, 465. 553
Meteoric stones, fall of 283, 457, 545
Mexico, on the diseases of 239
Milk, an ardent spirit distilled from 250
Moon, on the influence of the 85
Montpellier, prize questions at 455
Muriatic acid, lithontriptic power of 446
Muscular motion, on 180
National Institute, proceedings of the French 362
Naturalist's monthly report 189, 285, 372, 463, 551
Navy, on improving the medical department of the 442
Neander on the efficacy of tobacco 231
Needles, on the invention of aneurismal 32, 238
Nicotiana, various species of 120
Nipples, on sore 445
Nomenclature of the Pharmacopœia, on the 431
Norris, Mr. on tumours 165
Norway, horse-flesh eaten in 283
Oil, substitute for Florence 84
Olibanum, description of 160
Ophthalmia, two varieties of 357
——— Dr. Farrell on 522
Ornithology, account of the British 89, 286, 374
Oxyde, on the cystic 254
Oxygen, observations on 83
Oxymuriatic gas, experiments on 84
Paris, prize questions of the Agricultural Society at 455
Park, Mr. on vaccination 304
Parturition, case of unusual 205
Peake's, Mr. case of præternatural anus 449
Pearson's, Dr. observations on pus 326
Pepion, M. on urinary worms 544
Percy, Mr. on platina 509
Perforation of the glans penis, imperfection in the 41
Pharmacopœia, on the nomenclature of the London 431
Physicians, the college of, intention to publish their statutes 84
——— restrictions in the practice of 313
Placenta, on removing the 387
Platina, observations on 509
Plants, on the radicles of 452
Plumbi acetas, its efficacy in epilepsy 456
Poisons, on vegetable 361
——— on fish 398
Pole's, Dr. meteorological statements 142
Potass, incrystallizability of 456
——— on preparing the acetate of 518
Powders, on fulminating 457
Practice, on the division of medical 391
Prize awarded by the college of Surgeons 543
——— given by the French government 545
Psorophthalmy, description of 74
Puberty, case of premature 117
Puerperal fever, on an epidemic 193
Pupil, on the artificial 535
Pus, observations and experiments on 326
Quarrier, Mr. on fish poison 398
Queries on cancer 117
Questions, prize 454, 5
Radicles of plants, on the 452
Raleigh, Sir Walter, anecdotes of 121
Ramsden, Mr. reply to 41
——— explanation of 118
——— apology to 141
——— on diseases of the testicle 175, 265, 350
Registers, observations on meteorological 145
Remittents, observations on 4
Repletion, case of 475
Resins, on reddening litmus by 156
——— analysis of gum 320
Rheumatism, on the treatment of 82
Rhubarb, on the red colour of 499
Rhododendron chrysanthum, observations on 183
Ring, Mr. on the eau medicinale 207
——— on topical remedies 497
Ringwood cases, on the 307
Royal Society, proceedings of the 83, 180, 277, 361, 452
——— Edinburgh 181, 362
Rupture of the gall bladder, case of 185
Russel, Dr. on tobacco 135
Salter, Mr. on the improvement of grass lands 54
Sanguinaria Canadensis, properties of 166
Sarcocele of Egypt, on the 290
Savine in gout, on 544
Sauvages on the effects of stramonium 384
Scammony, analysis of 156
Scarlatina, observations on 35, 101, 406
——— on the treatment of 237, 299

Scirrhus testicle, on the 273
Sclerocele, practical observations on the 175
Sensation, theory of 467
Sense, on the abuse of the organs of 137
Serum, observations on white 150, 300
Shearman, Dr. on white lead 60
Small pox hospital, report of the 365
——— on infection from inoculated 490
——— subsequent to vaccination 390
Smith's, Mr. theory of sensation 467
Smithson, Mr. on the zeolite 361
Spasm, case of periodical 477
Staphyloma, observations on 161, 359
Stevenson, Mr. on a disease of the eye 67, 85
Stock's, Dr. life of Beddoes 91, 172, 259, 342
Stramonium, observations on 377, 509
Stones, fall of meteoric 283, 457, 545
Sugar in the serum of blood 277
Surgery, suggestions for the improvement of practical 146
——— on the practice of 394
Swallows, on the migration of 88
Syphilis, on the origin of 364
Tænia, on ol. tereb. in 546
Tape-worm, cure for the 23, 167
Taunton's Mr. case of obliterated iliac artery 35
Terry, Dr. on tænia 546
Testicle, on morbid enlargement of the 265, 350
Tetanus, case of 359
Tic douloureux, case of 167
Tobacco, on the history and use of 120, 319
——— origin of the word 479
Troops, on diseases of 13
Turpentine, its efficacy in tænia 167
Twin cases, on the management of 480, 485
Twin case, on an extraordinary 29
Twins, remarkable case of 311
——— on the practice in cases of 386
Urethra, membranous fence of the 41
Urinary worms, on 544
Uterus, case of excision of the 210
Vaccination at Edinburgh 460
——— Ceylon *ibid.*
——— in France, state of 181
——— addresses on 278, 459
——— at Liverpool 304
——— small pox subsequent to 390
——— at Glasgow 458
Vapour bath, on the 317
Vegetable poisons, on 361
Venereal disease, on the 81
Vertebræ, case of fractured 201
Vicarious menstruation, case of 24
Vinegar, test for sophisticated 85
Vitalis, Mr. on potass 456
Vomiting, case of obstinate 508
Voyage, on diseases in a long 17
Walcheren fever, report on the 1
Walden, Dr. on scarlatina 35
Walker, Mr. on the barometer 52, 149
——— Dr. on ophthalmia 357
Ward, Mr. two engravings of 85
Ware, Mr. on diseases of the eye 78, 161
Water, its effects on meat 252
Watt, Mr. on the aneurismal needle 238
Wernerian Natural History Society, meeting of the 453
Wilkinson, Mr. on the influence of the moon 85
Wishart's, Mr. case of fungus hæmatodes 448
Woollaston, Dr. on the cystic oxyde 254
——— on sugar in the blood 277
Worms, on urinary 544
Yellow fever, on the 360
Zeolite, description of the 361
Zoophyte, description of a 412

### REFERENCES TO THE PLATES.

Mr. TAUNTON's case of obliterated Iliac Artery - - Page 35
Mr. BROOKE's fossillized Remains of a Crocodile - - 97
Mr. LEWIS's Morbid Enlargement of the Clitoris - - - 136
Mr. EAGLAND's Truss for Exomphalos - - - 310
Dr. ANDREWS' case of enlarged lower Extremity - - 400

